# Genomic Epidemiology of *Vibrio cholerae* O1 Associated with Floods, Pakistan, 2010

**DOI:** 10.3201/eid2001.130428

**Published:** 2014-01

**Authors:** Muhammad Ali Shah, Ankur Mutreja, Nicholas Thomson, Stephen Baker, Julian Parkhill, Gordon Dougan, Habib Bokhari, Brendan W. Wren

**Affiliations:** COMSATS Institute of Information Technology, Islamabad, Pakistan (M.A. Shah, H. Bokhari);; Wellcome Trust Sanger Institute, Cambridge, UK (A. Mutreja, N. Thomson, J. Parkhill, G. Dougan);; Oxford University Clinical Research Unit, Ho Chi Mihn City, Vietnam (S. Baker);; London School of Hygiene and Tropical Medicine, London, UK (M.A. Shah, S. Baker, B.W. Wren)

**Keywords:** cholera, Pakistan, whole-genome sequencing, global phylogeny, Vibrio cholerae O1, floods, bacteria, single-nucleotide polymorphism

## Abstract

In August 2010, Pakistan experienced major floods and a subsequent cholera epidemic. To clarify the population dynamics and transmission of *Vibrio cholerae* in Pakistan, we sequenced the genomes of all *V. cholerae* O1 El Tor isolates and compared the sequences to a global collection of 146 *V. cholerae* strains. Within the global phylogeny, all isolates from Pakistan formed 2 new subclades (PSC-1 and PSC-2), lying in the third transmission wave of the seventh-pandemic lineage that could be distinguished by signature deletions and their antimicrobial susceptibilities. Geographically, PSC-1 isolates originated from the coast, whereas PSC-2 isolates originated from inland areas flooded by the Indus River. Single-nucleotide polymorphism accumulation analysis correlated river flow direction with the spread of PSC-2. We found at least 2 sources of cholera in Pakistan during the 2010 epidemic and illustrate the value of a global genomic data bank in contextualizing cholera outbreaks.

## Abstract 2

At least 2 subclades coexisted that had different antimicrobial-resistance profiles and patterns of spread.

In 2010, a surge in cholera cases seriously threatened public health across Pakistan, where previously sporadic cases of cholera had been reported (*1*–*3*). In late July and August 2010, record monsoon rainfall and the simultaneous glacier melt resulted in the worst flooding in the recorded history of Pakistan, affecting an area of 61,776 square miles and displacing >20 million persons (*4*). A cholera outbreak ensued, and the World Health Organization (WHO) reported 164 laboratory-confirmed cases with the help of National Institute of Health and other allied departments in Pakistan (*5*).

Despite the paucity of data on the impact of cholera in Pakistan before 2010, seasonal epidemics are known to have occurred every year since then. Cholera is endemic to South Asia (*6*) and the Bay of Bengal (*7*,*8*) and is spread through contaminated food and water, often after civil unrest or natural disasters*.* Pakistan is particularly at risk for waterborne disease because it is an agricultural economy with one of the most expansive water distribution systems in the world. This vast irrigation system depends largely on the Indus River, which originates on the northern slopes of the Kailash mountain range in India and runs north to south through the entire length of Pakistan with many tributaries, including the Zaskar, the Shyok, the Nubra, and the Hunza, converging in the northern region and flowing through the provinces of Ladakh, Baltistan, and Gilgit.

Not all *Vibrio cholerae* strains cause major disease outbreaks. Although *V. cholerae* has >200 serogroups, only serogroups O1 and O139 are associated with epidemics. Serogroup O1 isolates can be assigned to 2 biotypes, classical and El Tor; the latter is responsible for the current seventh pandemic that has spread in global radiations, or waves, originating in the Bay of Bengal (*8*). The clinical severity of cholera is associated with the production of cholera enterotoxin (CT), which is encoded by a gene on the 6.9-kb CTX prophage integrated within chromosome 1 of all pandemic *V. cholerae* O1 cholera strains (*9*). Historically, CTX prophages have been categorized as CTX^classical^ or CTX^El Tor^ on the basis of DNA sequence of the *rstR* and the sequence variation in *ctx*B gene. During the last 2 decades, new variants of El Tor biotypes have emerged and have been used to differentiate *V. cholerae* isolates (*10*). However, such approaches do not have the resolution required to stratify the highly clonal *V. cholerae* O1 isolates of the seventh pandemic sufficiently to understand their precise phylogeny and relate that to geographic distribution and spread.

The application of whole-genome sequence analyses has revolutionized our ability to resolve the *V. cholera*e O1 El Tor populations and more precisely determine the patterns of spread of cholera within the worst affected countries of the world. Clarifying the routes of spread of cholera in Pakistan provides the unprecedented opportunity to inform public health provision. This study showed that the 2010 cholera outbreak was, in fact, an epidemic within an epidemic explained by 2 independent introductions of cholera in the country, 1 from the south and 1 following the flood water as it moved from north to south along the Indus River.

## Materials and Methods

### Strain Collection

To determine whole-genome sequence type and single-nucleotide polymorphism (SNP)–based phylogeny analysis of *V. cholerae* following the 2010 floods in Pakistan, 38 *V. cholerae* O1 El Tor were isolated from fecal samples/rectal swabs of cholera patients during August–October in the flood-affected and -unaffected districts of Sindh, Khyber Pakhtunkhwa (KPK), and Punjab Provinces. Identification, serogroup, and biotype were determined by standard biochemical methods (*11*–*13*).

### Antimicrobial Susceptibility Test

The susceptibility of *V. cholerae* O1 El Tor to different antimicrobial drugs was tested by disk diffusion on MH agar. The antimicrobial drugs tested were ampicillin (10 μg), chloramphenicol (30 μg), ciprofloxacin (5 μg), cefotaxime (30 μg), ceftazidime (30 μg), erythromycin (15 μg), nalidixic acid (30 μg), ofloxacin (5 μg), streptomycin (10 μg), tetracycline (30 μg), trimethoprim (25 μg), and trimethoprim/sulfamethoxazole (1.25/23.71 μg). *Escherichia coli* ATCC25922 was used as quality control according to Clinical Laboratory Standards Institute guidelines (*14*). To interpret the results, we followed these guidelines. All antimicrobial drugs used during this study were purchased from Oxoid Limited (Hampshire, UK).

### Genome Sequencing

Unique index-tagged libraries, with 250-bp insertion size, were created and loaded on the 8 lanes in Illumina HiSeq cell (Illumina, San Diego, CA, USA) to perform 72-bp end sequencing of 96 separate libraries in each lane. After sequencing the index tags and libraries separately, the tag sequence information was used for assigning reads to the individual samples (*8*). All the samples achieved an average coverage of 200× in the regions where SNPs were called. All the data have been submitted to the European Nucleotide Archive with the accession codes.

### Whole-Genome Alignment and Detection of SNPs in the Core Genome

The 72-bp end read data obtained were mapped to El Tor reference strain N16961 (GenBank accession nos. AE003852 and AE003853) by using SMALT (www.sanger.ac.uk/resources/software/smalt) to obtain whole-genome alignment for all the strains in this study, and SNPs were called by using methods described by Harris et al. (*15*). Any unmapped reads and the sequences that were absent from N16961 reference genome were excluded from the core genome; thus, SNPs from these regions were not called. The SNPs called were filtered to remove the sites with a SNP quality score <30. SNPs that were absent in at least 75% of the reads at any heterogeneous mapped sites were excluded, and high-density SNP clusters or the possible recombination sites were excluded as described by Croucher et al. (*16*).

### Phylogenetic Analysis, Comparative Genomics, and Linear Regression Analysis

Default settings of RAxML version 0.7.4 (*17*) were used to estimate the phylogenetic trees on the basis of all the SNPs called from the genome as explained above. To calculate the number of SNPs on each branch, we reconstructed all the polymorphic events on the tree using PAML (*18*). M66, a pre–seventh-pandemic strain (accession nos. CP001233 and CP001234), was used as an outgroup to root the phylogenetic tree (*5*). The tree was visualized and ordered by using phylogenetic tree reading software, Figtree (http://tree.bio.ed.ac.uk/software/figtree/). The paired end reads were assembled by using a de novo genome assembly program Velvet version 0.7.03 (*19*), and a multicontig draft genome was generated for each sample. The parameters were set to give the best *k*-mer size and at least 20× *k*-mer coverage. To take advantage of the high similarity of the seventh-pandemic *V. cholerae* at the core genome, contigs were ordered by using Abacas with N16961 El Tor strain as reference (*20*). To each ordered draft genome, annotation transfer was made from the reference strain. Finally a genome comparison file was generated by TBLASTX (*21*) against N16961 FASTA sequence as a database to be used in Artemis Comparison Tool for manual comparison of the genomes (*22*). The final phylogenetic tree was opened by using Path-O-Gen version 1.3 (http://tree.bio.ed.ac.uk/software/pathogen), and the root-to-tip distance data for each strain were exported to Excel (Microsoft, Richmond, WA, USA). These data were used to plot a linear regression curve against date of isolation of the strain. The R^2^ correlation, slope, and p values were determined by using the inbuilt regression package of R statistical environment (www.r-project.org).

## Results

### Sample Collection

**Figure 1:**
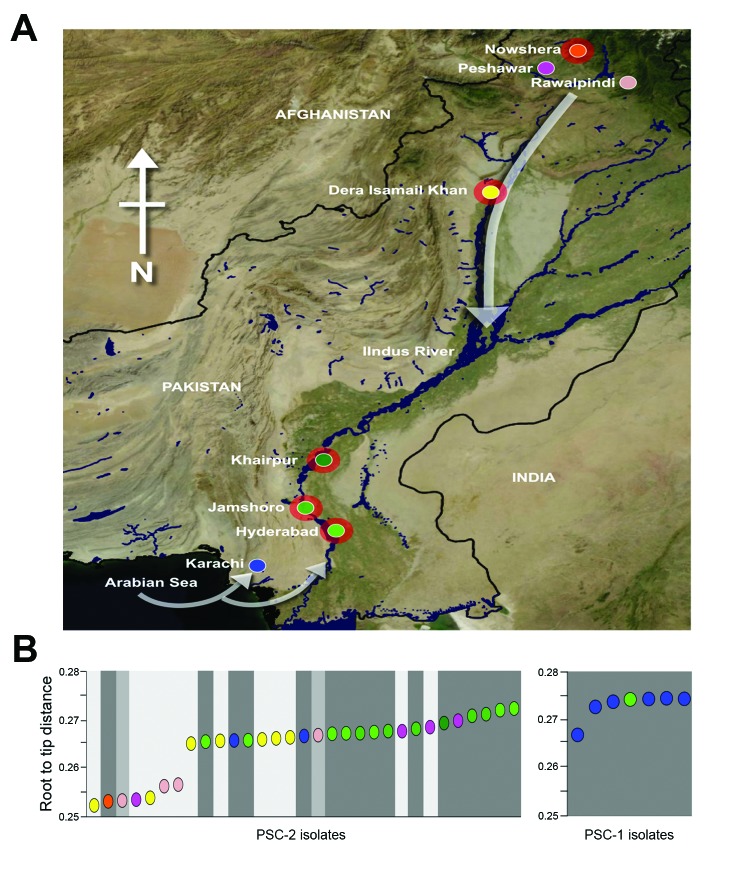
Cholera during the floods, Pakistan, 2010. A) North-oriented map of Pakistan indicating the 8 locations of *Vibrio cholerae* O1 El Tor isolation (shown by individual circles; red outer shading indicates the 5 locations that had flooding during the study period). White arrows indicate the hypothesized directions of spread of the various subclades of *V. cholerae* O1 El Tor. B) Cumulative root-to-tip distances of subclade 2 and subclade 1 *V. cholerae* O1 El Tor isolates. Each colored circle corresponds with an individual *V. cholerae* O1 El Tor isolate; colors correspond with the locations shown in panel A. Shading corresponds with the month of *V. cholerae* O1 El Tor isolation: light gray, August; medium gray, September; dark gray, October.

A total of 319 fecal samples were collected from patients who had acute diarrhea who reported in the KPK, Sindh, and Punjab Provinces during August–October 2010. Of these, 219 (69%) and 100 (31%) were from flood-affected and non–flood-affected regions, respectively (Table; Technical Appendix Table 1). A total of 38 *V. cholerae* O1 El Tor biotype (22 from flood-affected and 16 from non–flood-affected regions) were isolated and mapped to locations across Pakistan (Figure 1). The *V. cholerae* isolates were serogrouped and verified by PCR amplification of the *ompW* (*11*) and *rfb*O1/*rfb*O139 genes (*12*) (Technical Appendix Table 1). To interpret the progression of the outbreak, we used WHO data (year 2010, weeks 33–48 of the WHO epidemiologic reports in Pakistan) and compiled acute diarrhea incidence in Pakistan and its major provinces. These data demonstrated a dramatic increase in acute diarrhea cases in week 33, peaking in week 34 (fourth week of August) with ≈200,000 documented cases.

**Table:** Isolation of *Vibrio cholerae* O1 El Tor from flood-affected and non–flood-affected patients, Pakistan, 2010

Province, district	Flood status	Acute diarrhea samples collected, no. (%)	*V. cholerae* O1 El Tor isolates, no. (%)
Khyber Pakhtunkhwa			
Dera Ismail Khan	Affected	55	7 (13)
Nowshera	Affected	20	1 (5)
Charsada	Affected	40	0
Peshawar	Unaffected	50	4 (8)
Sindh			
Khairpur	Affected	36	1 (3)
Jamshoro	Affected	10	5 (50)
Sukkur	Affected	6	0
Shikarpur	Affected	6	0
Karachi	Unaffected	25	8 (32)
Hyderabad	Affected	19	8 (42)
Punjab			
Dera Ghazi Khan	Affected	2	0
Muzzaffargarh	Affected	25	0
Rawalpindi	Unaffected	25	4 (16)
Total, N = 319	Affected	219 (68)	22 (10)
	Unaffected	100 (32)	16 (7)

### Antimicrobial Susceptibility Patterns

Antimicrobial susceptibility testing by disk diffusion showed that all *V. cholerae* isolates were resistant to streptomycin, trimethoprim, trimethoprim/sulfamethoxazole, and nalidixic acid. All isolates from the flood-affected patients were susceptible to ciprofloxacin, ofloxacin, chloramphenicol, ampicillin, cefotaxime, ceftazidime, and tetracycline. However, 6 of 8 isolates from Karachi (a non–flood-affected coastal city) and 1 of 8 isolates from Hyderabad (located at the bank of Indus River) were tetracycline resistant. Five (63%) isolates from Karachi and 1 (13%) isolate from Hyderabad were also resistant to ceftazidime (Technical Appendix Table 2).

### Whole-Genome Phylogenetic Analysis

Whole-genome sequences of the 38 *V. cholerae* O1 strains were determined by using the Illumina sequencing platform. On the basis of genomewide SNPs, we constructed a high-resolution maximum-likelihood phylogenetic tree using previously described methods (*15*). Sequence reads were mapped to the reference genome sequence of *V. cholerae* O1 El Tor strain N16961 (isolated in Bangladesh in 1975, accession no. AE003852–3) and compared with 146 globally and temporally representative *V. cholerae* O1 El Tor strains (*8*).

**Figure 2:**
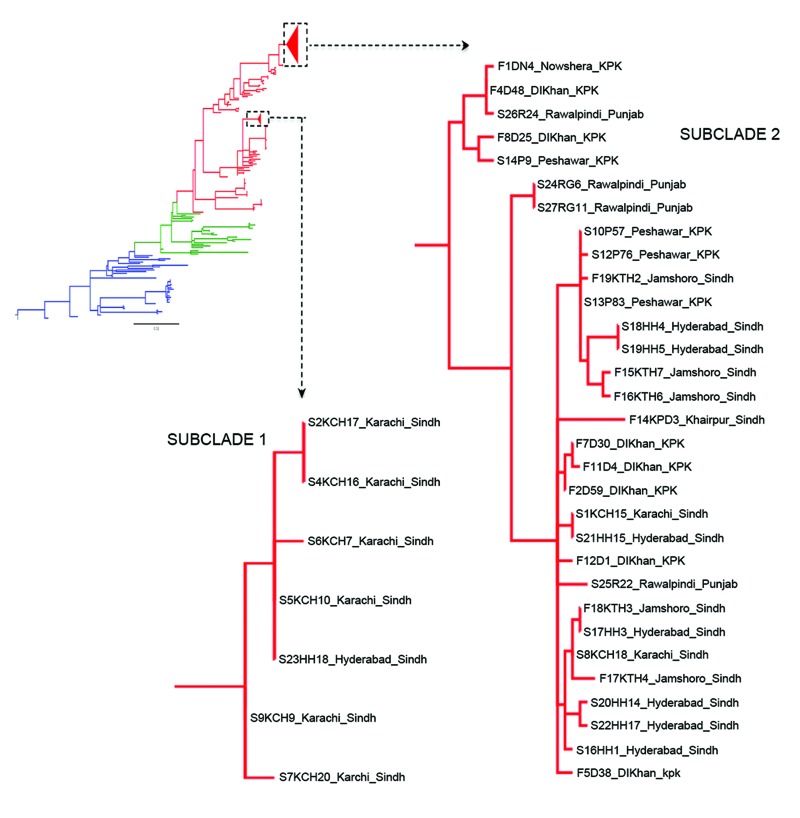
Phylogenetic tree showing the relative position of *Vibrio cholerae* O1 El Tor from Pakistan in wave 3 of the seventh-pandemic lineage, based on single-nucleotide polymorphism differences. The blue, green, and red branches represent waves 1, 2, and 3 respectively. The colors of the subclade 1 and 2 isolates match the colors assigned to the strain isolation locations in Figure 1, panel A.

**Figure 3:**
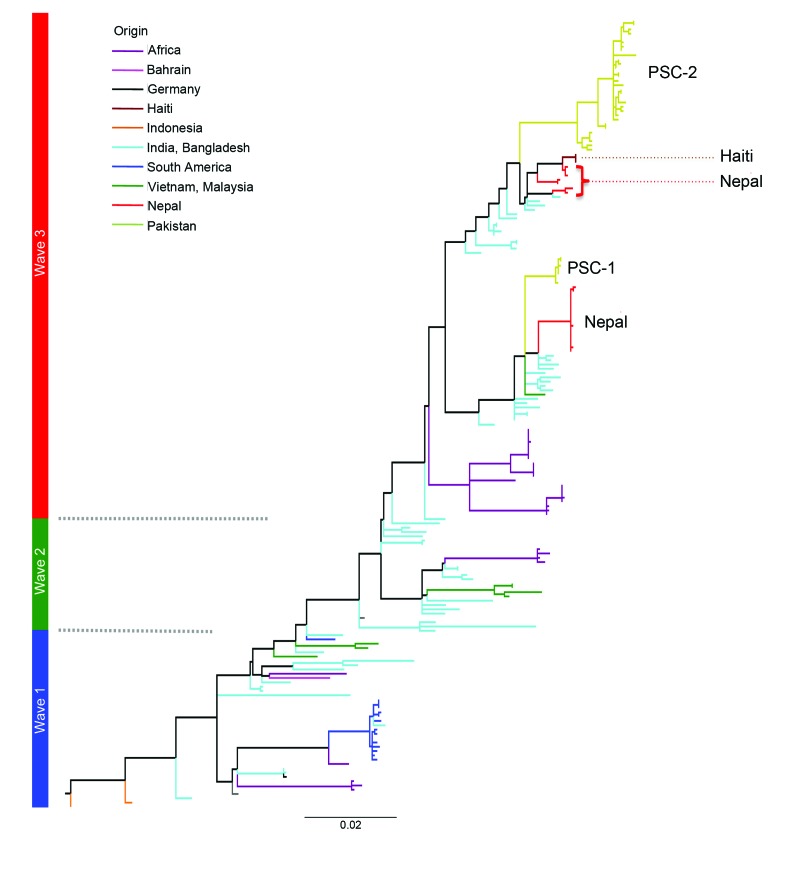
A single-nucleotide polymorphism–based maximum-likelihood phylogeny showing the position of *Vibrio cholerae* O1 El Tor from Pakistan in wave 3 of the seventh-pandemic lineage relative to the Haiti and Nepal strains of Hendriksen et al. (*23*). Waves 1, 2, and 3 are labeled in blue, green, and red respectively. Scale bar indicates substitutions per variable site.

The consensus tree showed that all Pakistan strains fell within 2 contemporary subclades (PSC-1 and PSC-2), both of which branched from different positions within the third transmission wave of the seventh-pandemic lineage (Figures 2, 3). The PSC-1 isolates were derived from cases located in the coastal city of Karachi (6/7) and Hyderabad (1/8), whereas the PSC-2 isolates originated from a wide geographic region comprising flood-affected and non–flood-affected inland regions (30/31) and 1 case from Karachi (Figure 1). After removal of genomic recombination sites, 1,826 variable genomic sites defined variation in the El Tor global phylogeny, and PSC-1 and PSC-2 had only 12 and 22 distinguishing SNPs from their third-wave ancestors, respectively (Figure 2). Within each subclade, the strains were very closely related, with only 4 SNPs within the PSC-1 isolates and 76 SNPs within the PSC-2 isolates.

We previously showed that genomic variation occurs in the seventh-pandemic El Tor *V. cholerae* at a clock-like rate (*8*). Consequently, we plotted root-to-tip distances of the PSC-1 and PSC-2 isolates against isolation date and geographic location. We observed a strong and statistically significant phylogeographic correlation between mutation rate and isolation date, recognizing that the PSC-1 and PSC-2 strains isolated earlier were closer to the root of the Pakistan clades, whereas those collected later were further away (R^2^ = 0.27, p<0.001). The SNP acquisition rate in the Pakistan isolates occurred at 0.288 SNPs/month (3.4 SNPs/year) and was in accordance with our estimations inferred from a global seventh-pandemic strain collection (3.3 SNPs/year) (Technical Appendix Figure 1).

Furthermore, we observed that the earlier isolates of PSC-2 clade, displaying shorter root-to-tip distances, were in closer proximity to the source of the Indus River and were mainly isolated from Peshawar, Nowshera, Rawalpindi, and Dera Ismail Khan in the north of Pakistan. Conversely, isolates in October tended to be from the southern regions of Khairpur, Jamshoro, Hyderabad, and Karachi (Figure 1, panel B). A root-to-tip phylogenetic tree distance plot of the PSC-2 subclade against distance from the source of the Indus River confirmed this association (R^2^ = 0.35, p<0.001; Technical Appendix Figure 2). The observed pattern is consistent with the origins and progression of the floods, which began in Peshawar in late July and followed the course of the Indus River southwest, passing Nowshera, Dera Ismail Khan, Khairpur, Jamshoro, and Hyderabad in August.

### Subclade Signature Deletions

The Pakistan subclades could be distinguished from other El Tor *V. cholerae* by subclade-specific deletions in the DNA sequences, particularly in the 2, Vibrio pathogenicity island–1 and Vibrio seventh pandemic−2 (VPI-1 and VSP-2), that impact on the relative transmissibility and virulence of the respective subclades. All the PSC-1 isolates had a unique 3-gene deletion in the VPI-1 (VC_0819–0821) and a 4-gene deletion within the VSP-2 (VC_0495–0498), which was previously identified in El Tor strains responsible for outbreaks in Bangladesh in 2008 (*23*). In contrast, the PSC-2 isolates had an 18-gene deletion (VC_0495–0512) in VSP-2 comparable to some of the most recently characterized strains of El Tor *V. cholerae* O1, including those from the Haiti outbreak in 2010 (*23*). VP1-I is intact in PSC-2 isolates except for a frame-shift mutation in the accessory colonization factor gene, *acfC* (VC_0841).

## Discussion

We used whole-genome SNP-based analyses of *V. cholerae* from areas of Pakistan affected by the major floods of 2010 to assign isolates onto the seventh-pandemic *V. cholerae* O1 El Tor phylogenetic tree (*7*). All isolates mapped to wave 3 of the current pandemic as 2 distinct subclades, PSC-1 and PSC-2, in general agreement with their time of isolation. Both Pakistan subclades are located on the tree close to other isolates of South Asia origin, including Nepal, as well as those from the recent outbreak in Haiti (*23*) (Figure 3). However, the genomic analysis clearly shows that both Pakistan clades represent distinct outbreaks not directly related to *V. cholerae* isolated elsewhere. The geographic distribution of the isolates in PSC-1 and PSC-2 is revealing. Isolates from PSC-1 are largely limited to the non–flood-affected coastal city of Karachi, and only 1 PSC-1 isolate was from the nearby city of Hyderabad, whereas isolates from PSC-2 were from inland flood- and non–flood-affected areas countrywide (Figure 1).

Our root-to-tip distance analyses shows a correlation between the direction of the flow of the Indus River and the spread of *V. cholerae* and suggests that during the floods, 2 or possibly 3 routes of cholera spread in Pakistan. The most parsimonious explanation for the spread of PSC-2 isolates throughout Pakistan was that they followed the river along with the floodwater. In contrast, PSC-1 isolates appear to have originated in the coastal region of Pakistan, and they failed to penetrate far inland. The third possible route is represented by sporadic cholera cases in PSC-2 caused by the flood-associated isolates in areas not affected by the floodwaters but by travel of infected persons.

From our comparative genomics analysis, we identified signature deletions in the VPI-1 and VSP-2 genomic islands. PSC-1 has a unique 3-gene deletion in VPI-1, which includes *aldA* (aldehyde dehydrogenase), *tagA* (a mucinase), and a predicted coding sequence encoding a hypothetical protein. *tagA* plays a role in host cell surface modification (*24*), and its deletion may affect the virulence and transmission of the PSC-1 isolates. To our knowledge, this deletion has not been previously reported; however, the deletion of entire VPI-1 was reported in an isolate from a patient in the United States who had traveled to Pakistan (*25*). A 4-gene deletion common to PSC-1 in VSP-2 (VC-0495–498) was similar to those found in isolates from cholera outbreaks in Bangladesh in 2008 (*23*). The 18-gene deletion common to PSC-2 isolates in VSP-2 is similar to deletions found in El Tor isolates from cholera outbreaks in Haiti 2010 and from southeastern China in 2005 (*23*,*26*) because PSC-2 is closer to the Haiti 2010 cholera strains on the phylogenetic map (Figure 3). A gene located in this deletion encodes a putative type IV pilin, which may affect the colonization and virulence potential of these strains. These deletions are consistent with the position of these subclades on the phylogenetic tree of *V. cholerae* O1 El Tor. However, it is perhaps surprising that elements within the 2 pathogenicity islands appear dispensable in both PSC-1 and PSC-2 isolates. The relative impact of these deletions on *V. cholerae* pathogenesis and relative transmissibility remains to be evaluated.

The genetic organization of the SXT locus, which encodes resistance to trimethoprim, sulfamethoxazole, and streptomycin, is similar in both subclades, and as expected, all isolates were resistant to these drugs. In addition, all isolates were resistant to nalidixic acid and had intact VC_1577 (*almG*), VC_1578 (*almF*), and VC_1579 (*almE*) genes, which have recently been shown to explain the genetic basis for resistance to polymixin B/nalidixic acid (*27*). Overall PSC-1 isolates were resistant to more antimicrobial drugs than were SC-2 isolates. For example, all PSC-1 isolates were resistant to tetracycline, whereas all PSC-2 isolates were susceptible. However, *V. cholerae* O1 of both PSCs are sensitive to ciprofloxacin, except 1 from Hyderabad. Therefore, we believe that cholera in Pakistan can effectively be treated by a single dose of ciprofloxacin rather than by a repeatedly higher number of doses of erythromycin (*28*), which may be useful in preventing epidemics during natural disasters.

The position of the PSC-1 and PSC-2 isolates on the global phylogenetic tree of *V. cholerae* O1 places them close to strains from India isolated in 2006 and 2007 and strains from Bangladesh and India isolated in 2004 and 2005, respectively (*8*) (Figure 2). PSC-1 appears to be the most recent emergent subclone, with isolates mainly from the coastal port city of Karachi. Therefore, we speculate that PSC-1 might have been introduced into Pakistan at this time, either by an unknown route to sites close to the sea or, perhaps more likely, directly from the marine/estuarine ecosystem or might be related to travel into this region.

By contrast, the wider distribution of PSC-2 isolates throughout Pakistan suggests that at the time of sampling this was the major El Tor subclade affecting the country. It is likely that PSC-2 spread through Pakistan predominantly by the flood water because the genetically older isolates were found nearer the source of the river, whereas genetically newer isolates were found further downstream closer to the river outflow into the Arabian Sea. However, a few isolates do not fit the linear regression curve of root-to-tip distance with time. These anomalous isolates might be explained by human travel among different provinces in Pakistan. The phylogeny of the 2 subclades shows unequivocally that PSC-1 and PSC-2 have evolved from 2 different recent ancestors. Thus, during the floods, at least 2 subclades of *V. cholerae* coexisted in Pakistan with different antimicrobial-resistance profiles and patterns of spread: an epidemic within an epidemic.

Technical Appendix Phenotypic and genotypic characterization of antimicrobial resistance, scatter plot of root-to-tip distance vs. date of isolation for PSC-1 and PSC-2 combined, and scatter plot of root-to-tip distance vs. distance from river source for PSC-2.
