# Supplementary material for: Genomic Epidemiology of Vibrio cholerae O1 Associated with Floods, Pakistan, 2010
Source: Emerg Infect Dis. 2014 Jan;20(1):13–20. doi: 10.3201/eid2001.130428 (PMC3884714; doi:10.3201/eid2001.130428)
Supplement: Technical Appendix — Phenotypic and genotypic characterization of antimicrobial resistance, scatter plot of root-to-tip distance vs. date of isolation for PSC-1 and PSC-2 combined, and scatter plot of root-to-tip distance vs. distance from river source for PSC-2. [file 13-0428-Techapp-s1.pdf]

Article DOI: http://dx.doi.org/10.3201/eid2001.130428

Genomic Epidemiology of *Vibrio cholerae* O1 associated with Floods, Pakistan, 2010

Technical Appendix

Technical Appendix Table 1. Phenotypic and genotypic characterization of antimicrobial resistance

District	*V. cholerae* O1 El Tor isolates, no.	Antimicrobial resistance profile	Genotypic characterization of antimicrobial resistance			
			TMP/SXT	*sul1/sul2*	*strB*	*dfrA1/dfrA18*
Dera Ismail Khan†	07	STR, TMP/SXT,TMP, NA	+	–/+	+	+/–
Nowshera†	01	STR, TMP/SXT, TMP, NA	+	–/+	+	+/–
Peshawar‡	04	STR, TMP/SXT, TMP, NA	+	–/+	+	+/–
Khairpur†	01	STR, TMP/SXT, TMP, NA	+	–/+	+	+/–
Jamshoro†	04	STR, TMP/SXT, TMP, NA	+	–/+	+	+/–
	01	STR, TMP/SXT, TMP, NA, CHL	+	–/+	+	+/–
Hyderabad†	03	STR, TMP/SXT, TMP, NA	+	–/+	+	+/–
	01	STR, TMP/SXT, TMP, NA, CIP	+	–/+	+	+/–
	01	STR, TMP/SXT, TMP, NA, AMP, CHL	+	–/+	+	+/–
	01	STR, TMP/SXT, TMP, NA, CTX	+	–/+	+	+/–
	01	STR, TMP/SXT, TMP, NA, CAZ	+	–/+	+	+/–
	01	STR, TMP/SXT, TMP, NA, TET	+	–/+	+	+/–
Karachi‡	03	STR, TMP/SXT, TMP, NA, TET, CAZ	+	–/+	+	+/–
	02	STR, TMP/SXT, TMP, NA, TET	+	–/+	+	+/–
	02	STR, TMP/SXT, TMP, NA, TET, CAZ, AMP	+	–/+	+	+/–
	01	STR, TMP/SXT, TMP, NA	+	–/+	+	+/–
Rawalpindi‡	04	STR, TMP/SXT, TMP, NA	+	–/+	+	+/–

*TMP/SXT, trimethoprim/sulfamethoxazole; +, positive; -, negative; STR, streptomycin; TMP, trimethoprim; NA, nalidixic acid; CHL, chloramphenicol; CIP, ciprofloxacin; AMP, ampicillin; CTX, cefotaxime; CAZ, ceftazidime; TET, tetracycline.
†Flood-affected.
‡Flood unaffected.

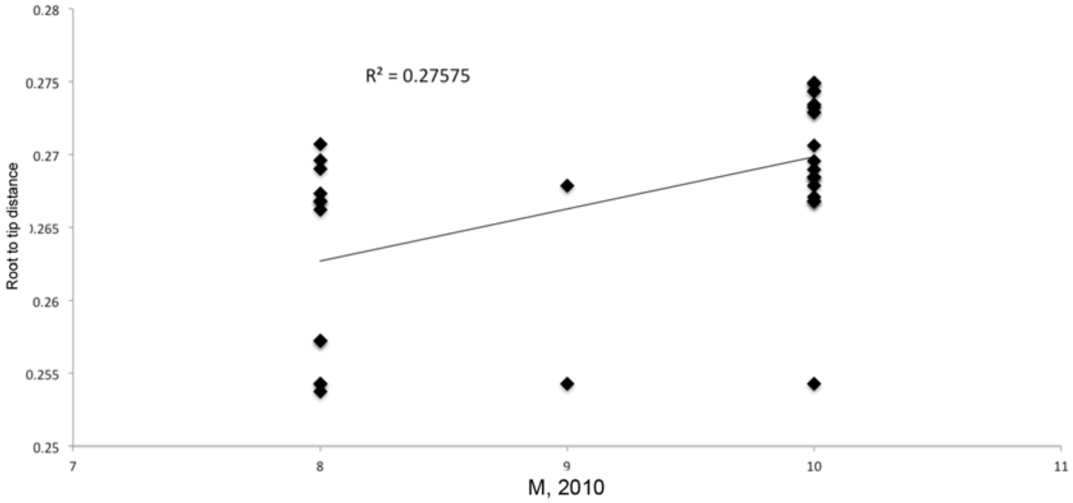

Technical Appendix Figure 1. Scatter plot of root-to-tip distance vs. date of isolation for PSC-1 and PSC-2 combined.

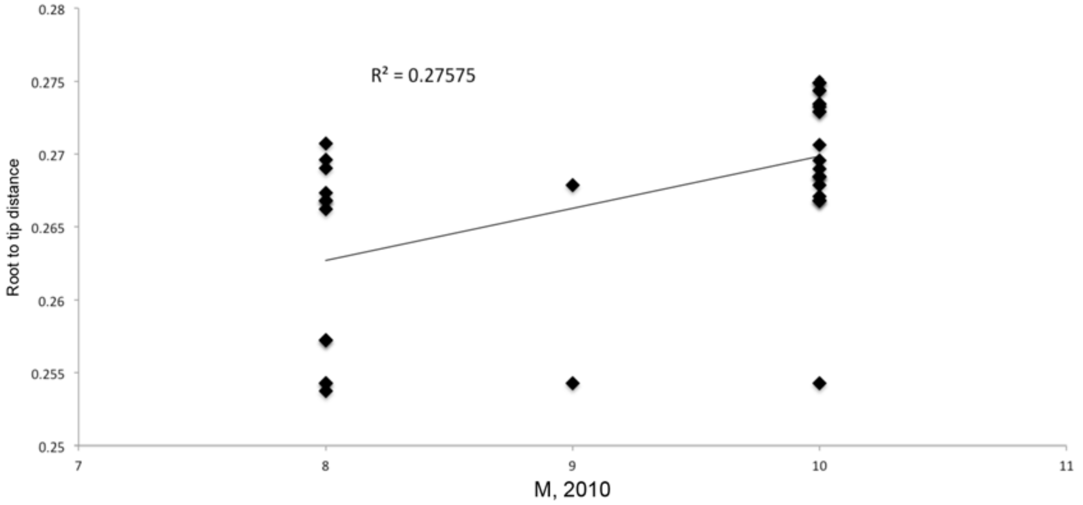

Technical Appendix Figure 2. Scatter plot of root-to-tip distance vs. distance from river source for PSC-2.
